# Author Correction: IL-12 reprograms CAR-expressing natural killer T cells to long-lived Th1-polarized cells with potent antitumor activity

**DOI:** 10.1038/s41467-024-49582-6

**Published:** 2024-06-18

**Authors:** Elisa Landoni, Mark G. Woodcock, Gabriel Barragan, Gabriele Casirati, Vincenzo Cinella, Simone Stucchi, Leah M. Flick, Tracy A. Withers, Hanna Hudson, Giulia Casorati, Paolo Dellabona, Pietro Genovese, Barbara Savoldo, Leonid S. Metelitsa, Gianpietro Dotti

**Affiliations:** 1 Lineberger Comprehensive Cancer Center, University of North Carolina, Chapel Hill, NC USA; 2 Division of Oncology, Department of Medicine, University of North Carolina, Chapel Hill, NC USA; 3 Center for Advanced Innate Cell Therapy, Texas Children’s Cancer Center, Department of Pediatrics, Baylor College of Medicine, Houston, TX USA; 4 Dana-Farber/Boston Children’s Cancer and Blood Disorder Center, Boston, USA; 5 Harvard Medical School, Boston, USA; 6 Experimental Immunology Unit, Division of Immunology, Transplantation and Infectious Diseases, IRCCS San Raffaele Scientific Institute, Milan, Italy; 7 Department of Pediatrics, University of North Carolina, Chapel Hill, NC USA; 8 Department of Microbiology and Immunology, University of North Carolina, Chapel Hill, NC USA

**Keywords:** Cancer immunotherapy, Interleukins, Tumour immunology

Correction to: *Nature Communications* 10.1038/s41467-023-44310-y, published online 02 January 2024

In this article the affiliation details for Giulia Casorati and Paolo Dellabona were incorrectly given as ‘Experimental Immunology Unit, Division of Immunology, Transplantation and Infectious Diseases, San Raffaele Scientific Institute, Milan, Italy’ but should have been ‘Experimental Immunology Unit, Division of Immunology, Transplantation and Infectious Diseases, IRCCS San Raffaele Scientific Institute, Milan, Italy’. The original article has been corrected.

