# Supplementary material for: IL-12 reprograms CAR-expressing natural killer T cells to long-lived Th1-polarized cells with potent antitumor activity
Source: Nat Commun. 2024 Jan 2;15:89. doi: 10.1038/s41467-023-44310-y (PMC10762263; doi:10.1038/s41467-023-44310-y)
Supplement: Supplementary file 1 — Supplementary Information [file 41467_2023_44310_MOESM1_ESM.pdf]

IL-12 Reprograms CAR-Expressing Natural Killer T cells to Long-Lived Th1-Polarized Cells with Potent Antitumor Activity

Figure S1

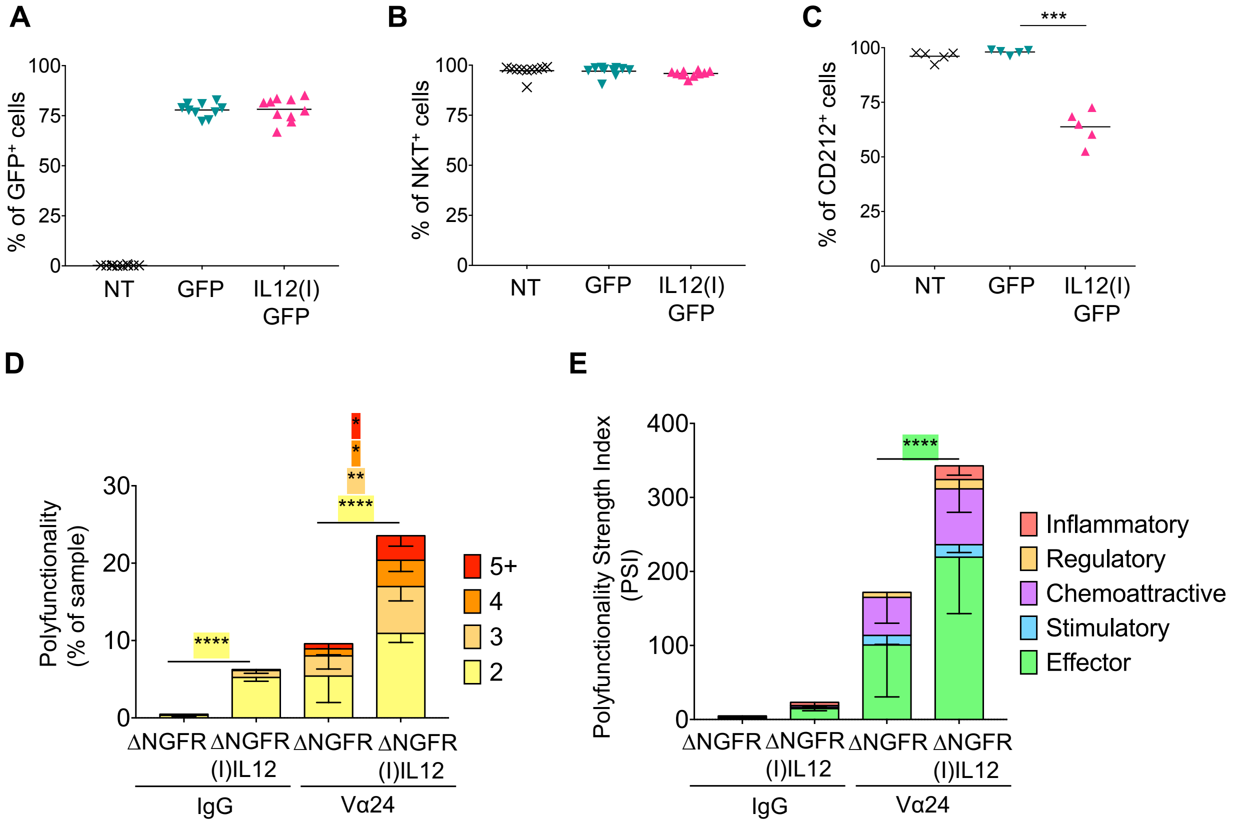

Figure S1. NKTs transduced with the IL12(I)GFP vector are polyfunctional. A. Summary of the NKT transduction efficiency measured as percentage of GFP$^+$ cells in non-transduced NKTs (NT), NKTs transduced with the control GFP vector (GFP) and with the IL12(I)GFP vector. Mean is shown, n = 10. Source data are provided as a Source Data file. **B**. Summary of the NKT purity in NT, GFP and IL12(I)GFP NKTs at day 14 of culture. Mean is shown, n = 10. Source data are provided as a Source Data file. **C.** Summary of the IL-12 receptor expression measured as percentage of CD212$^+$ cells in NT, GFP and IL12(I)GFP NKTs. Mean is shown, n = 5; ***p=0.0007; paired T test. Source data are provided as a Source Data file. **D.** NKTs were transduced with retroviral vectors in which GFP was switched with ΔNGFR to allow sorting of the transduced NKTs[1]. Single-cell polyfunctionality of ΔNGFR and IL12(I)ΔNGFR NKTs after activation with the control IgG Ab or the iNKT Ab (Vα24) was determined. Mean and standard deviation are shown, n = 4. IgG ****p<0.0001; Vα24 ****p<0.0001; **p=0.0014; 4 cytokines *p=0.0319; 5+ cytokines *p=0.0316; 2way ANOVA. Source data are provided as a Source Data file. **E.** Single-cell PSI of ΔNGFR and IL12(I)ΔNGFR NKTs after activation with the control IgG Ab or the iNKT Ab (Vα24). Mean and standard deviation are shown, n = 4. ****p<0.0001; 2way ANOVA. Source data are provided as a Source Data file.

Figure S2

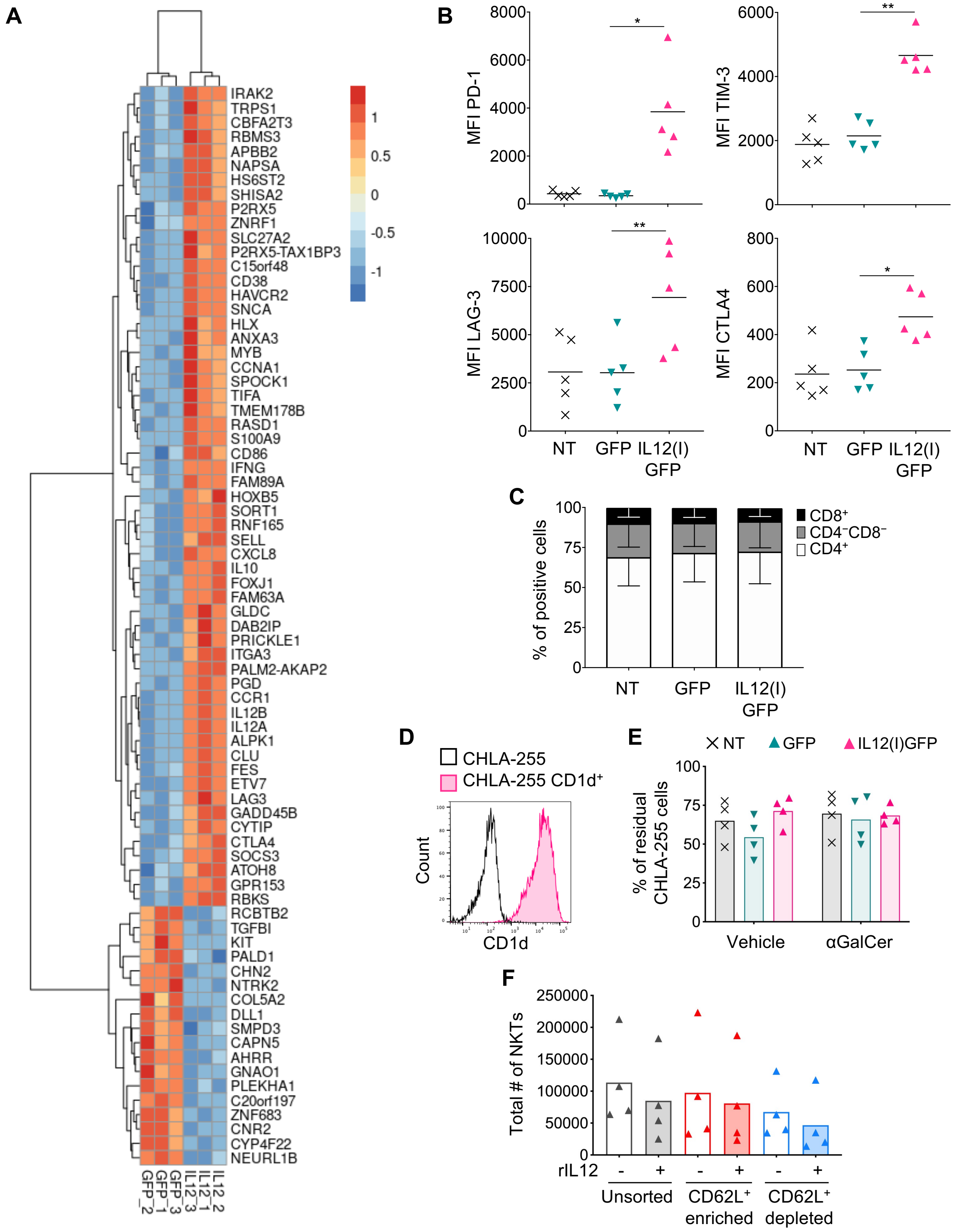

A
IRAK2
TRPS1
CBFA2T3
RBMS3
APBB2
NAPSA
HS6ST2
SHISA2
P2RX5
ZNRF1
SLC27A2
P2RX5-TAX1BP3
C15orf48
CD38
HAVCR2
SNCA
HLX
ANXA3
MYB
CCNA1
SPOCK1
TIFA
TMEM178B
RASD1
S100A9
CD86
IFNG
FAM89A
HOXB5
SORT1
RNF165
SELL
CXCL8
IL10
FOXJ1
FAM63A
GLDC
DAB2IP
PRICKLE1
ITGA3
PALM2-AKAP2
PGD
CCR1
IL12B
IL12A
ALPK1
CLU
FES
ETV7
LAG3
GADD45B
CYTIP
CTLA4
SOCS3
ATOH8
GPR153
RBKS
RCBTB2
TGFBI
KIT
PALD1
CHN2
NTRK2
COL5A2
DLL1
SMPD3
CAPN5
AHRR
GNAO1
PLEKHA1
C20orf197
ZNF683
CNR2
CYP4F22
NEURL1B
GFP_2
GFP_1
GFP_3
IL12_3
IL12_1
IL12_2
1
0.5
0
-0.5
-1
B
MFI PD-1
MFI TIM-3
MFI LAG-3
MFI CTLA4
NT
GFP
IL12(I) GFP
C
% of positive cells
CD8+
CD4−CD8−
CD4+
NT
GFP
IL12(I) GFP
D
CHLA-255
CHLA-255 CD1d+
Count
CD1d
E
NT
GFP
IL12(I)GFP
% of residual CHLA-255 cells
Vehicle
αGalCer
F
Total # of NKTs
rIL12
Unsorted
CD62L+ enriched
CD62L+ depleted

Figure S2. IL-12 reprograms NKTs to activated and proliferative cells. A. Heat map showing the comparison of the RNAseq data of GFP vs IL-12(I)GFP NKTs. **B.** Summary of the Mean Fluorescence Intensity (MFI) of PD-1, TIM-3, LAG-3, and CTLA4 in NT, GFP, and IL12(I)GFP NKTs at day 14 of culture. Mean is shown, n = 5; *p=0.0132 for PD-1; **p=0.0031 for TIM-3; **p=0.0057 for LAG-3; *p=0.0154 for CTLA4; paired T test. Source data are provided as a Source Data file. **C.** Summary of the quantification of CD4 single positive, CD8 single positive, or CD4 and CD8 double negative NKTs in NT, GFP, and IL12(I)GFP NKTs at day 14 of culture. Mean and standard deviation are shown, n = 10. Source data are provided as a Source Data file. **D.** Representative flow cytometry plot of the CD1d expression in wild-type and CD1d-transduced CHLA-255 cells. **E.** Summary of the quantification of residual tumor cells when NKTs were cocultured with wild type CHLA-255 cells with or without αGalCer (100 ng/ml). Four days later cells were collected and stained with the anti-iTCR (Vβ11) and anti-GD2 Abs to identify NKTs and neuroblastoma cells, respectively, by flow cytometry. Mean is shown, n = 4. Source data are provided as a Source Data file. **F.** Non-sorted NKTs, CD62L^{+} sorted NKTs and CD62L depleted NKTs were cultured for 6 days with or without recombinant human IL-12 (10 ng/ml). Data show cell counts at day 6; mean is shown; n = 4. Source data are provided as a Source Data file.

Figure S3

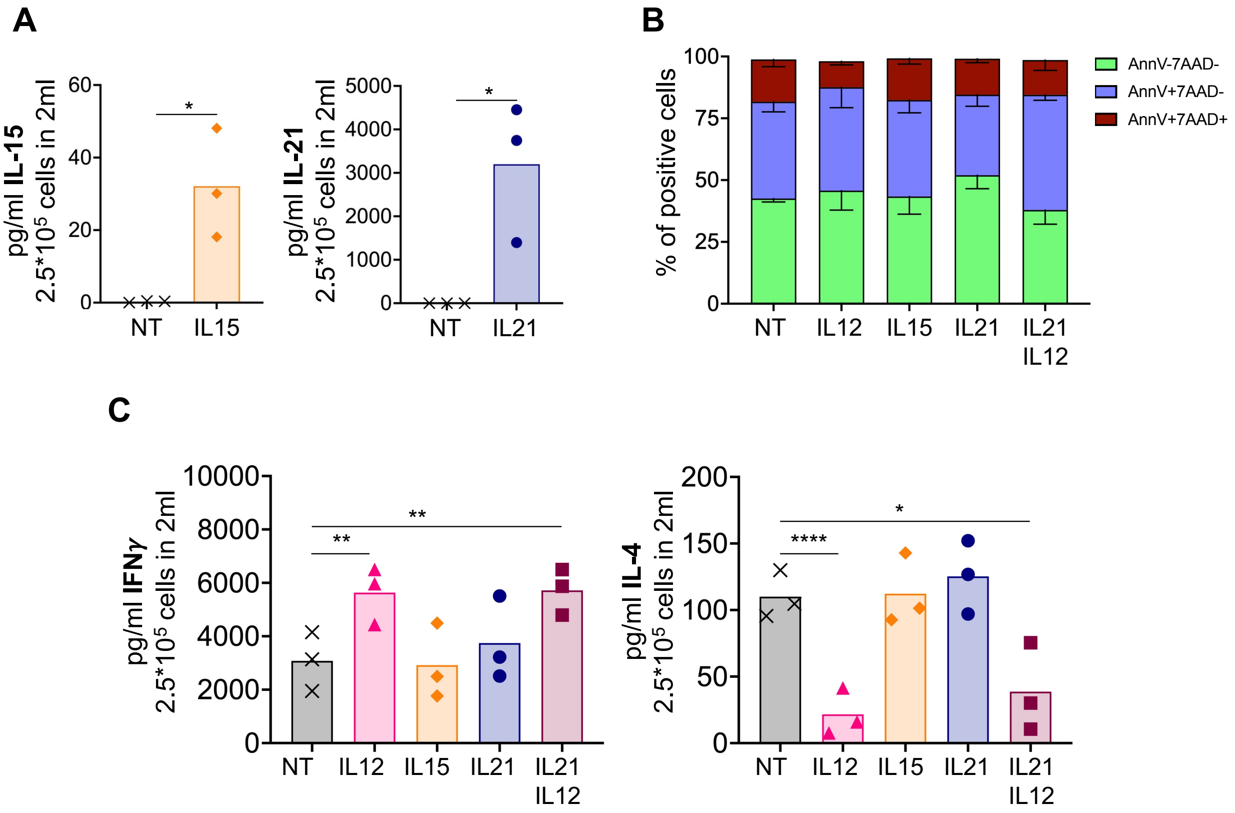

Figure S3. IL-15 and IL-21 do not cause Th1 polarization of NKTs. NKTs were genetically engineered to express either IL-15 or IL-21 and compared to NKTs expressing IL-12. **A**. Quantification of human IL-15, and IL-21 in supernatants collected 48 and 24 hrs after activation of control NKTs (NT) and IL-15 or IL-21 transduced NKTs activated with the iNKT Ab (1 x 10^5 cells/well in 24 well plates in 2 mL of complete media without cytokines). Mean is shown, n = 3; *p=0.0217 for IL-15; *p=0.0258; unpaired t test. Source data are provided as a Source Data file. **B.** Summary of the AnnexinV$^-$/7-AAD$^-$, AnnexinV$^+$/7-AAD$^-$ or AnnexinV$^+$/7-AAD$^+$ cells in NKTs (NT) and IL-12, IL-15, IL-21 or IL-12/IL-21 transduced NKTs after activation with the iNKT Ab for 48 hrs. Mean and standard deviation are shown, n = 3. Source data are provided as a Source Data file. **C.** Quantification of IFN-γ, and IL-4 in supernatants collected 24 hrs after activation with the iNKT Ab of NKTs (NT) and IL-12, IL-15, IL-21 or IL-12/IL-21 transduced NKTs. Mean is shown, n = 3; * p=0.0376 ; **p=0.0069 NT vs IL-12; **p=0.0072 NT vs IL-21+IL-12; ****p<0.0001; one way ANOVA. Source data are provided as a Source Data file.

Figure S4

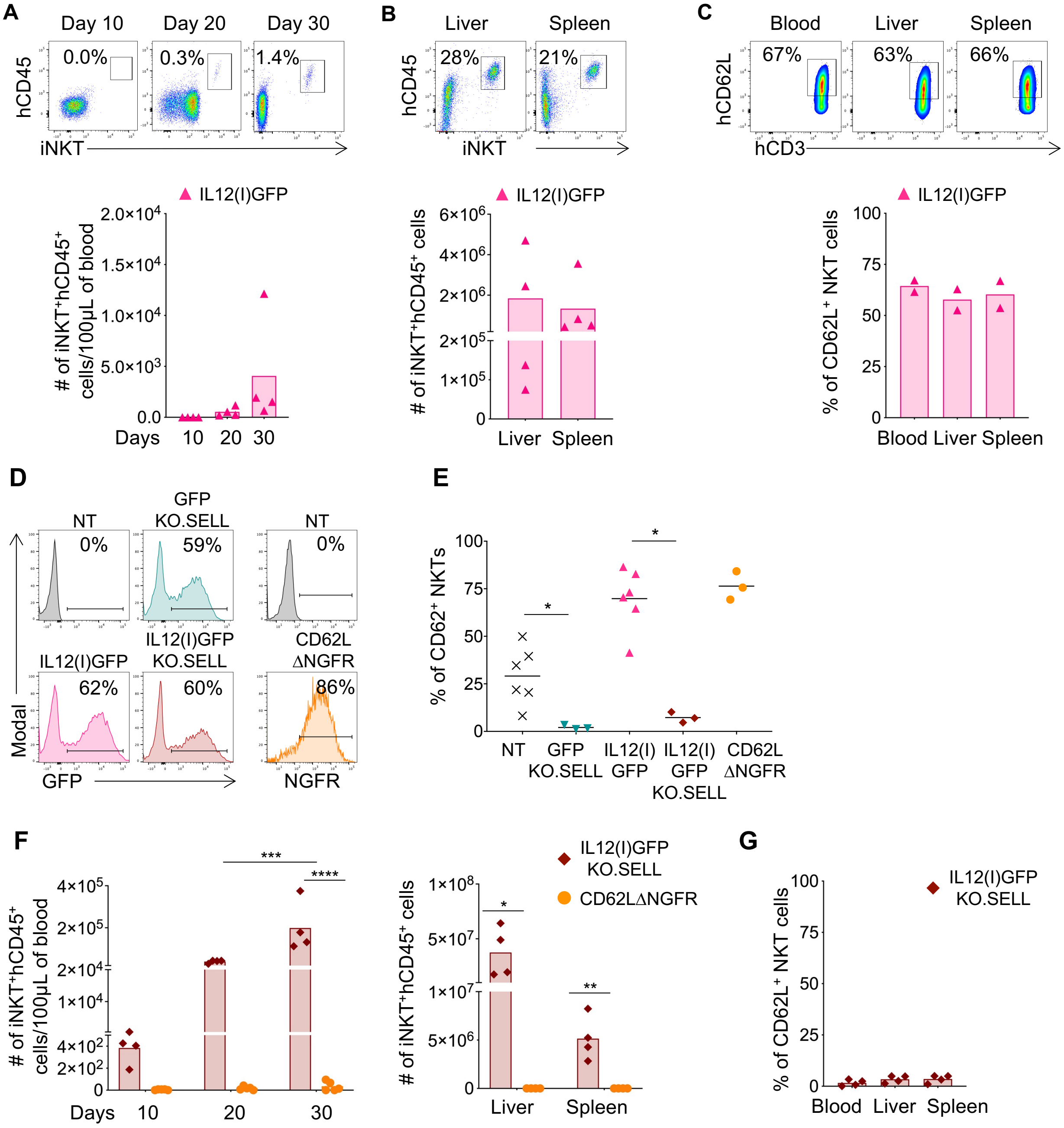
A
Day 10
Day 20
Day 30
0.0%
0.3%
1.4%
hCD45
iNKT
B
Liver
Spleen
28%
21%
hCD45
iNKT
C
Blood
Liver
Spleen
67%
63%
66%
hCD62L
hCD3
IL12(I)GFP
# of iNKT+hCD45+ cells/100μL of blood
Days 10 20 30
# of iNKT+hCD45+ cells
Liver Spleen
% of CD62L+ NKT cells
Blood Liver Spleen
D
NT 0%
GFP KO.SELL 59%
NT 0%
IL12(I)GFP 62%
IL12(I)GFP KO.SELL 60%
CD62L ΔNGFR 86%
Modal
GFP
NGFR
E
% of CD62+ NKTs
NT
GFP KO.SELL
IL12(I) GFP
IL12(I) GFP KO.SELL
CD62L ΔNGFR
F
# of iNKT+hCD45+ cells/100μL of blood
Days 10 20 30
IL12(I)GFP KO.SELL
CD62LΔNGFR
# of iNKT+hCD45+ cells
Liver Spleen
G
IL12(I)GFP KO.SELL
% of CD62L+ NKT cells
Blood Liver Spleen

Figure S4. IL-12 confers long-term longevity *in vivo* to NKTs. A. Representative flow cytometry plots (upper panel) and summary (lower panel) of the quantification of human NKTs ($iNKT^+CD45^+$) in peripheral blood samples collected 10, 20, and 30 days after the infusion of IL12(I)GFP NKTs in of non-tumor-bearing NSG mice in the re-transplant model. Mean is shown, n = 4, from two independent experiments**.** Source data are provided as a Source Data file. **B**. Representative flow cytometry plots (upper panel) and summary (lower panel) of the quantification of human NKTs ($iNKT^+CD45^+$) in livers and spleens collected at sacrifice (day 30 - 50) from non-tumor-bearing NSG mice infused with IL12(I)GFP NKTs described in A. Mean is shown, n = 4, from two independent experiments. Source data are provided as a Source Data file. **C.** Representative flow cytometry plots (upper panel) and summary (lower panel) showing the expression of CD62L in IL12(I)GFP NKTs at sacrifice in peripheral blood, livers, and spleens of mice described in A. Mean is shown, n = 2. Source data are provided as a Source Data file. **D.** Representative flow cytometry plots of the GFP or NGFR expression in transduced NKTs; n = 3. **E.** Summary of the quantification of $CD62L^+$ NKTs in NT, GFP with the knockout of CD62L (GFP KO.SELL), IL12(I)GFP, and IL12(I)GFP with the knockout of CD62L (IL12(I)GFP KO.SELL) and CD62L overexpressing (CD62L.ΔNGFR) NKTs at day 14 of culture. Mean is shown, n = 3 for GFP KO.SELL, IL12(I)GFP KO.SELL and CD62L.ΔNGFR; n = 6 for NT and IL12(I)GFP. **p*=0.0462 NT vs. GFP KO.SELL; **p*=0.0370 IL12(I)GFP vs. IL12(I)GFP KO.SELL; paired t test. Source data are provided as a Source Data file. **F.** Summary of the quantification of human NKTs ($iNKT^+CD45^+$) in peripheral blood samples collected 10, 20, and 30 days after the infusion of IL12(I)GFP KO.SELL or CD62L.ΔNGFR NKTs, and in livers and spleens at sacrifice. Mean is shown, n = 4, from two independent experiments; **p*=0.0167; **p*=0.0041; ****p*=0.0002; *****p*<0.0001; two-way ANOVA with Bonferroni correction. Source data are provided as a Source Data file. **G.** Summary of the expression of CD62L in IL12(I)GFP KO.SELL NKTs at sacrifice in the peripheral blood, livers, and spleens. Mean is shown, n = 4, from two independent experiments. Source data are provided as a Source Data file.

Figure S5

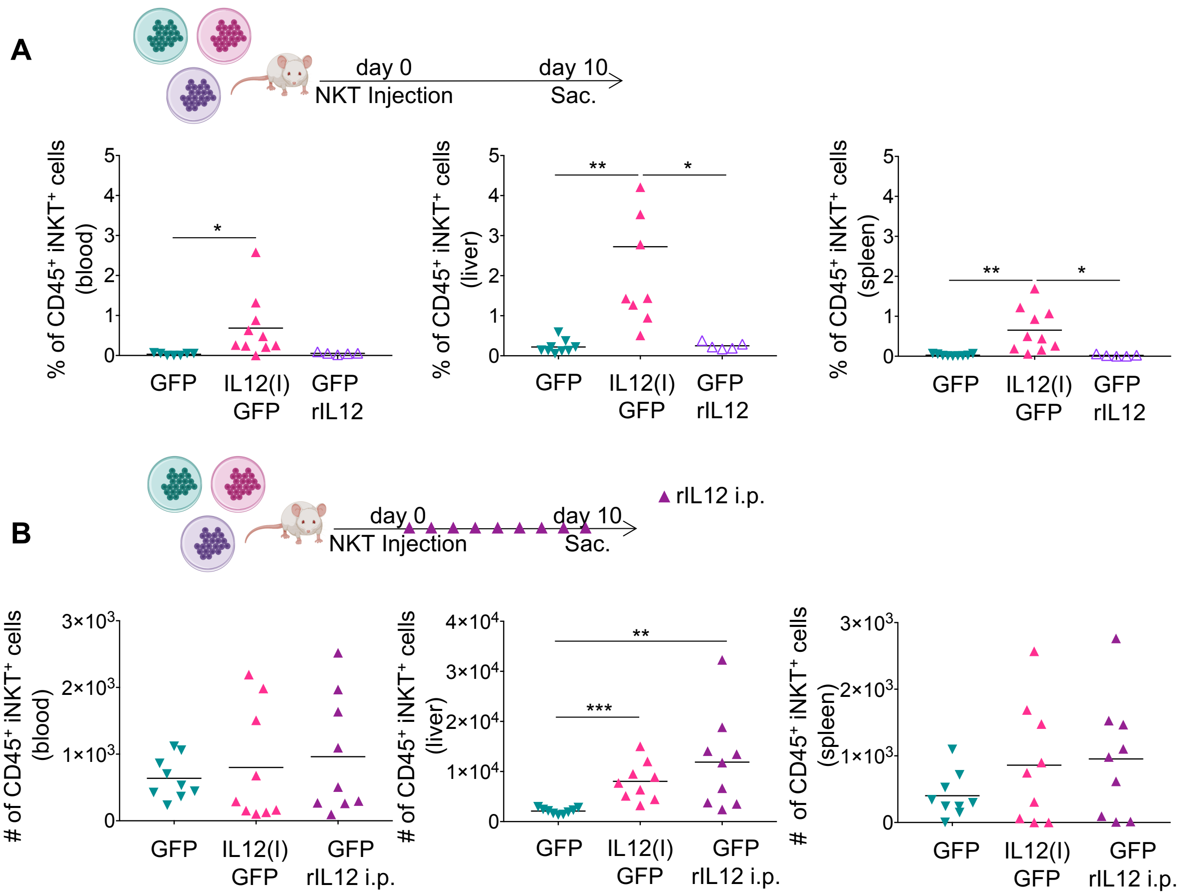

Figure S5. IL-12 continuous exposure is required to ensure the long-term engraftment of NKTs *in vivo*. A. NSG non-tumor-bearing mice were engrafted i.v. with GFP NKTs, IL-12(I)GFP NKTs or GFP NKTs cultured *ex vivo* with 10 ng/ml of human recombinant IL-12. At day 10 mice were sacrificed, and peripheral blood, livers, and spleens were collected to quantify human NKTs (iNKT^{+}CD45^{+}). Mean is shown, n = 7 for GFP, n = 10 for IL12(I)GFP and n = 5 for GFP rIL12i.p; *p=0.0418 in peripheral blood; *p=0.0135 and **p=0.0011 in livers; *p=0.0248 and **p=0.0033 in spleens; unpaired t test. Source data are provided as a Source Data file. **B.** NSG non-tumor-bearing mice were engrafted i.v. with GFP NKTs, IL-12(I)GFP NKTs, or GFP NKTs cultured *ex vivo* with 10 ng/ml of human recombinant IL-12. Mice also received 0.5 µg of recombinant IL-12 administered intraperitoneally daily. On day 10 mice were sacrificed, and the peripheral blood, livers, and spleens were collected to quantify human NKTs (iNKT^{+}CD45^{+}). Mean is shown, n = 9; **p=0.0072; ***p=0.0003; unpaired T test. Source data are provided as a Source Data file.

Figure S6

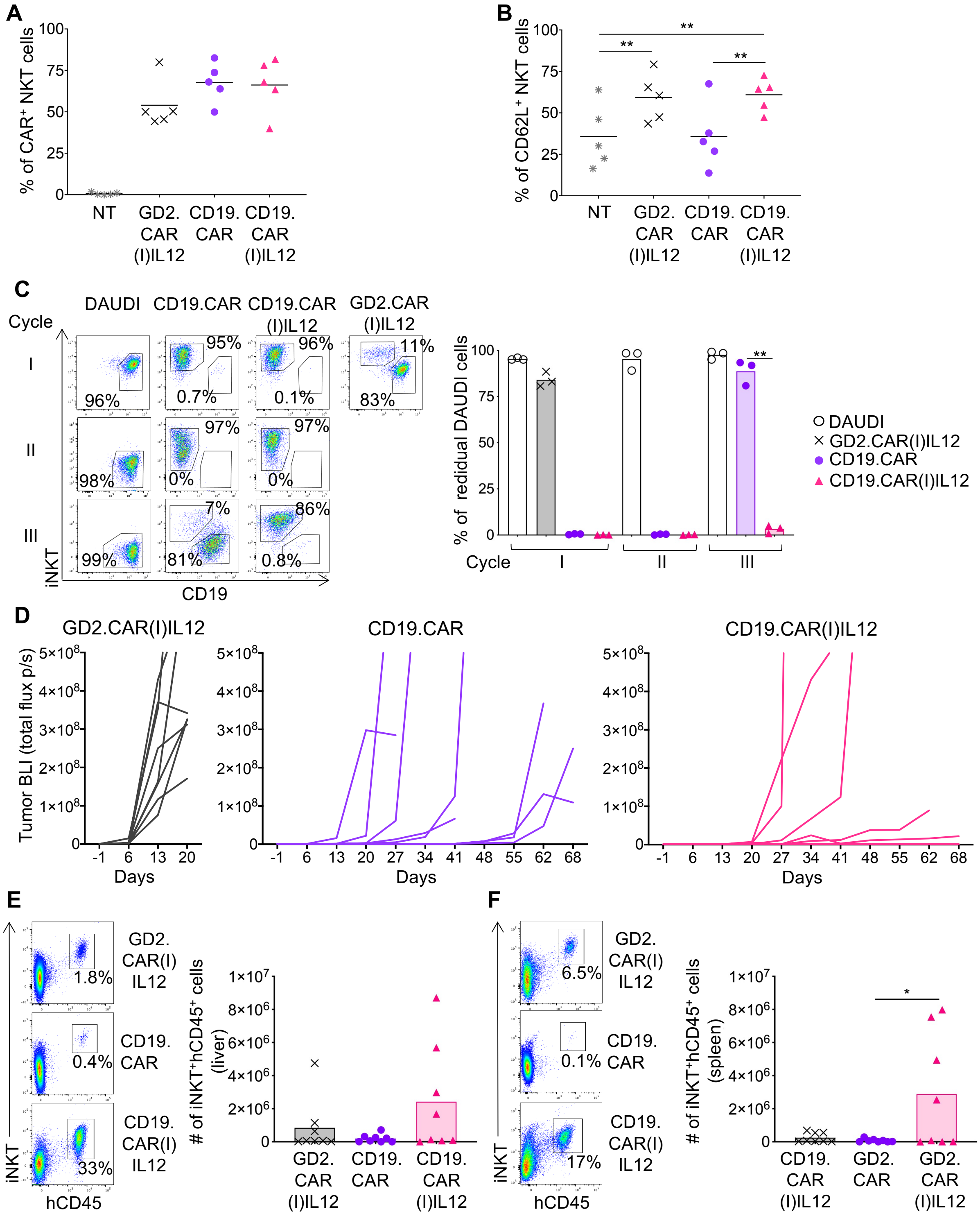
A
% of CAR+ NKT cells
NT
GD2. CAR (I)IL12
CD19. CAR
CD19. CAR (I)IL12
B
% of CD62L+ NKT cells
**
C
DAUDI
CD19.CAR
CD19.CAR (I)IL12
GD2.CAR (I)IL12
Cycle
I
II
III
iNKT
CD19
% of residual DAUDI cells
DAUDI
GD2.CAR(I)IL12
CD19.CAR
CD19.CAR(I)IL12
D
GD2.CAR(I)IL12
CD19.CAR
CD19.CAR(I)IL12
Tumor BLI (total flux p/s)
Days
E
iNKT
hCD45
# of iNKT+hCD45+ cells (liver)
F
# of iNKT+hCD45+ cells (spleen)

Figure S6. Antitumor activity of CD19.CAR-NKTs expressing IL-12. A. Summary of CAR expression in control (NT) and transduced NKTs assessed at day 14 of culture. Mean is shown, n = 5. Source data are provided as a Source Data file. **B.** Summary of CD62L expression in NT and transduced NKTs assessed at day 14 of culture. Mean is shown, n = 5; ***p*=0.0017 NT vs. GD2.CAR(I)IL12; ***p*=0.0082 CD19.CAR vs. CD19.CAR(I)IL12; ***p*=0.0074 NT vs. CD19.CAR(I)IL12; paired t test. Source data are provided as a Source Data file. **C.** Representative flow cytometry plots (left) and summary (right) of the quantification of residual tumor cells after each cycle when NKTs were cocultured with Daudi cells (E:T=1:2). Cells were collected and stained with iNKT and scFv-specific CD19 Abs to identify NKTs and lymphoma cells, respectively, by flow cytometry. Mean is shown, n = 3; ***p*=0.0010, paired t test. Source data are provided as a Source Data file. **D.** BLI tumor kinetics; n = 8 from three independent experiments. Source data are provided as a Source Data file. **E.** Quantification of human NKTs ($iNKT^+CD45^+$) in livers collected at the time of sacrifice. Mean is shown, n = 8, from three independent experiments. Source data are provided as a Source Data file. **F.** Quantification of human NKTs ($iNKT^+CD45^+$) in spleens collected at the time of sacrifice. Mean is shown, n = 8, from three independent experiments; **p*=0.0383; unpaired t test. Source data are provided as a Source Data file.

Figure S7

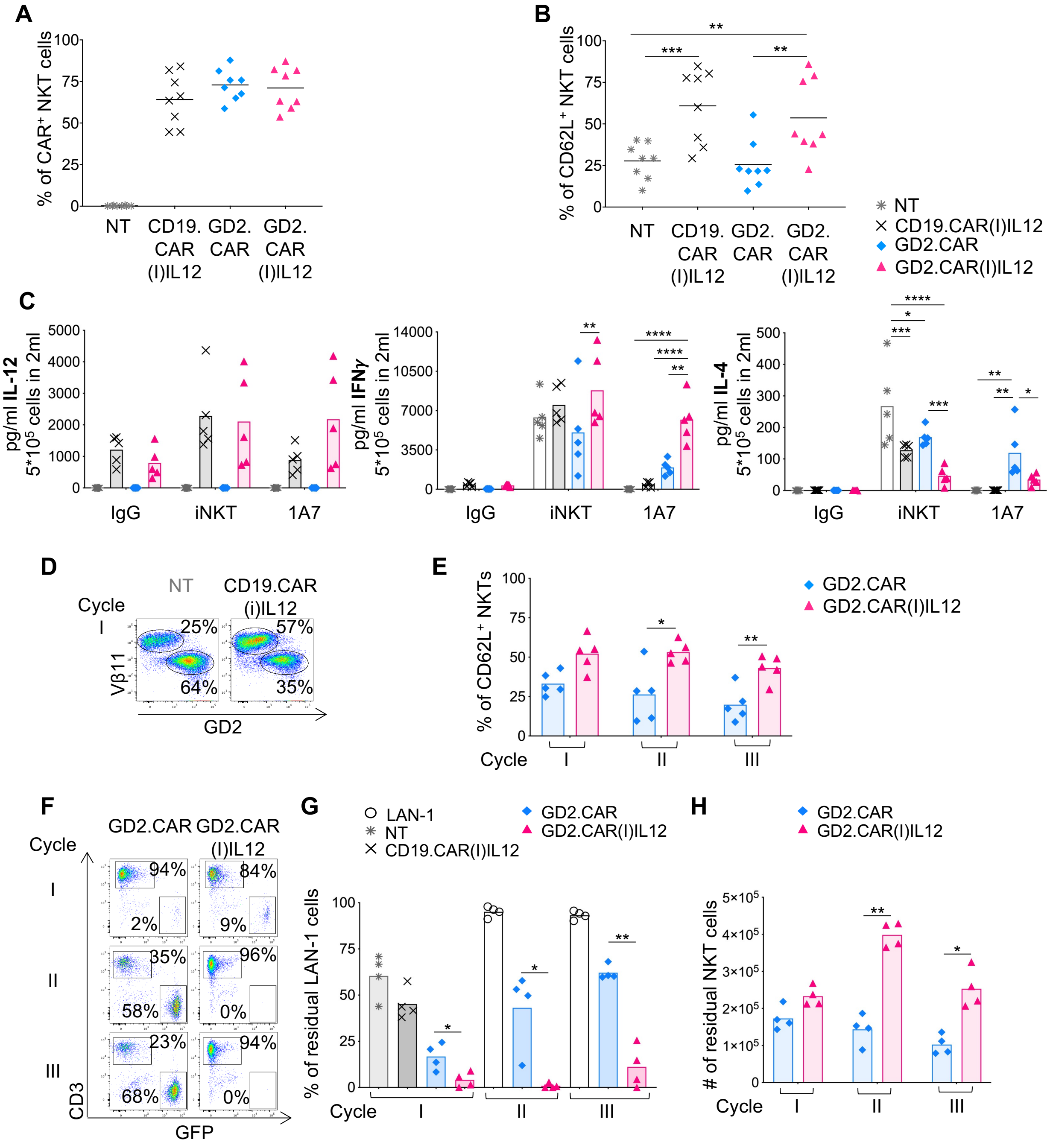

A
% of CAR+ NKT cells
NT
CD19. CAR (I)IL12
GD2. CAR
GD2. CAR (I)IL12
B
% of CD62L+ NKT cells
NT
CD19.CAR(I)IL12
GD2.CAR
GD2.CAR(I)IL12
C
pg/ml IL-12 5*10^5 cells in 2ml
pg/ml IFNγ 5*10^5 cells in 2ml
pg/ml IL-4 5*10^5 cells in 2ml
IgG
iNKT
1A7
D
NT
CD19.CAR (i)IL12
Cycle I
Vβ11
GD2
E
% of CD62L+ NKTs
GD2.CAR
GD2.CAR(I)IL12
Cycle
F
GD2.CAR
GD2.CAR (I)IL12
Cycle
CD3
GFP
G
LAN-1
NT
CD19.CAR(I)IL12
GD2.CAR
GD2.CAR(I)IL12
% of residual LAN-1 cells
Cycle
H
GD2.CAR
GD2.CAR(I)IL12
# of residual NKT cells
Cycle

Figure S7. Antitumor activity of GD2.CAR-NKTs expressing IL-12. A. Summary of CAR expression in control (NT) and transduced NKTs assessed at day 14 of culture. Mean is shown, n = 8. Source data are provided as a Source Data file. **B.** Summary of CD62L expression in NT and transduced NKTs assessed at day 14 of culture. Mean is shown, n = 8; ***p*=0.0024 NT vs. GD2.CAR(I)IL12; ***p*=0.0013 GD2.CAR vs. GD2.CAR(I)IL12; ****p*=0.0009 NT vs. CD19.CAR(I)IL12; paired t test. Source data are provided as a Source Data file. **C.** Quantification of human IL-12, IFN-γ, and IL-4 in supernatants collected 24 hrs after activation of NT, CD19.CAR(I)IL12, GD2.CAR and GD2.CAR(I)IL12 with the iNKT or 1A7 Abs. Isotype control (IgG) was used as negative control. Cytokines were measured in supernatants collected 24 hrs after plating 5 x 10^5 cells/well in 24 well plates in 2 mL of complete media without cytokines. Mean is shown, n = 5; **p*<0.0388; ***p*<0.0084; ****p*<0.0009; *****p*<0.0001; two-way ANOVA. Source data are provided as a Source Data file. **D.** Representative flow cytometry plots of residual tumor cells after the first cycle when NKTs were cocultured with CHLA-255 (E:T=1:1) and then collected and stained with iTCR (Vβ11) and GD2 Abs to identify NKTs and neuroblastoma cells, respectively, by flow cytometry. Source data are provided as a Source Data file. **E**. Quantification of the CD62L expression at the end of each cycle when GD2.CAR and GD2.CAR(I)IL12 NKTs were cocultured with CHLA-255 cells. Mean is shown, n = 5; **p*=0.0119; ***p*=0.0082; paired t test. **F-H.** Representative flow cytometry plots (**F**), summary of the quantification of residual tumor cells (**G**) and NKTs (**H**) after each cycle when NKTs were cocultured with LAN-1 (GFP^+) cells (E:T=1:2) and then collected and stained with CD3 to identify NKTs by flow cytometry. Mean is shown, n = 4; cycle I tumor cell % **p*=0.0140; cycle II tumor cell % **p*=0.0267; cycle III tumor cell % ***p*=0.0058; cycle II NKT number ***p*=0.027; cycle III NKT number **p*=0.0252; paired t test. Source data are provided as a Source Data file.

Figure S8

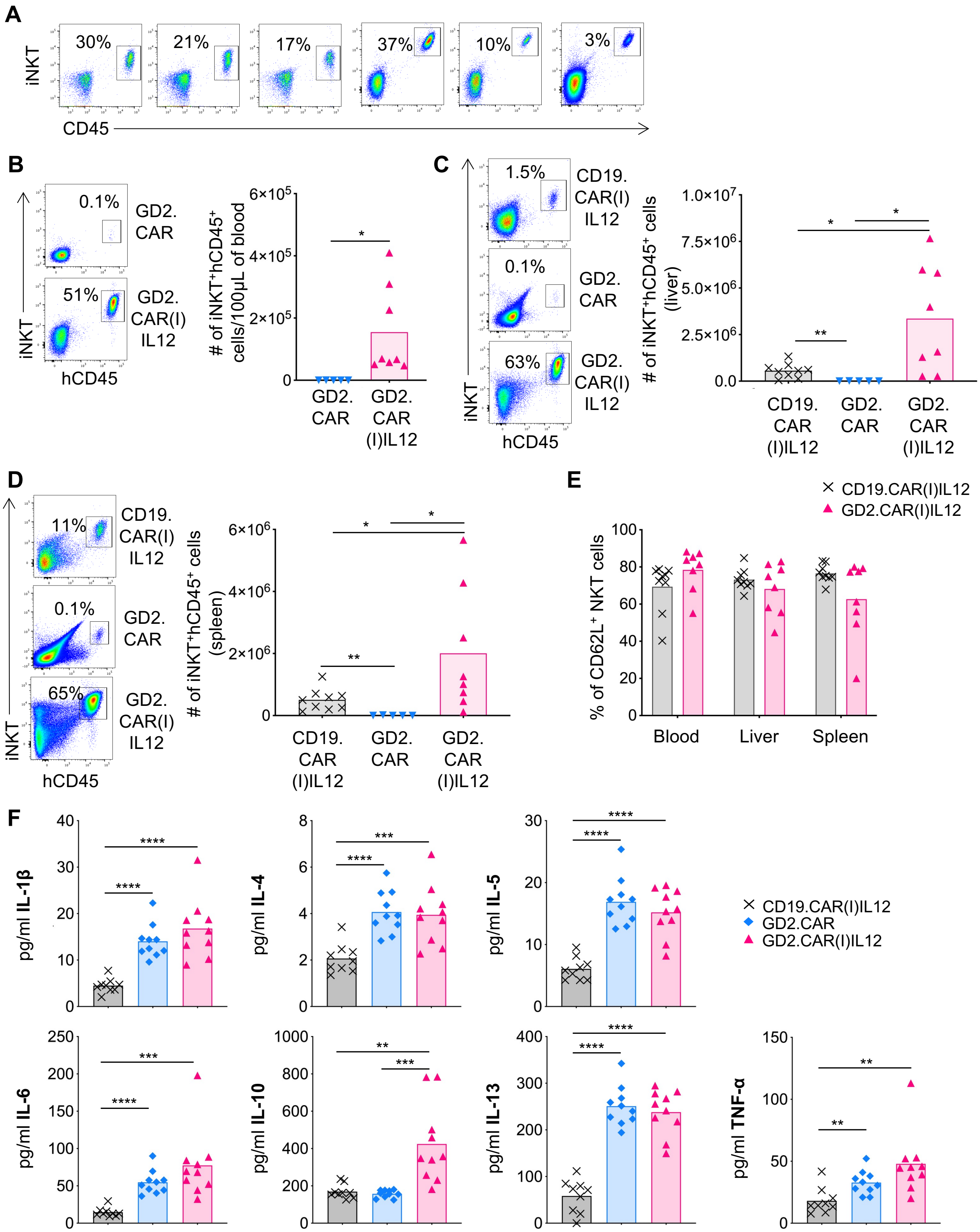

A
iNKT
CD45
30%
21%
17%
37%
10%
3%
B
iNKT
hCD45
0.1%
GD2.
CAR
51%
GD2.
CAR(I)
IL12
# of iNKT+hCD45+ cells/100μL of blood
GD2. CAR
GD2. CAR (I)IL12
C
iNKT
hCD45
1.5%
CD19. CAR(I) IL12
0.1%
GD2. CAR
63%
GD2. CAR(I) IL12
# of iNKT+hCD45+ cells (liver)
CD19. CAR (I)IL12
GD2. CAR
GD2. CAR (I)IL12
D
iNKT
hCD45
11%
CD19. CAR(I) IL12
0.1%
GD2. CAR
65%
GD2. CAR(I) IL12
# of iNKT+hCD45+ cells (spleen)
E
CD19.CAR(I)IL12
GD2.CAR(I)IL12
% of CD62L+ NKT cells
Blood
Liver
Spleen
F
pg/ml IL-1β
pg/ml IL-4
pg/ml IL-5
CD19.CAR(I)IL12
GD2.CAR
GD2.CAR(I)IL12
pg/ml IL-6
pg/ml IL-10
pg/ml IL-13
pg/ml TNF-α

Figure S8. GD2.CAR(I)IL12 NKTs persist long-term *in vivo* in a metastatic neuroblastoma tumor model. A. Flow cytometry plots showing the percentage of human NKTs (iNKT$^+$CD45$^+$) in peripheral blood samples collected 8 weeks after the infusion of GD2.CAR(I)IL12 NKTs in three independent experiments. **B.** Quantification of human NKTs (iNKT$^+$CD45$^+$) in peripheral blood samples collected at the time of sacrifice. Mean is shown, n = 5 for GD2.CAR and n = 8 for GD2.CAR(I)IL12, from three independent experiments; **p*=0.0354, unpaired t test. Source data are provided as a Source Data file. **C.** Quantification of human NKTs (iNKT$^+$CD45$^+$) in livers collected at the time of sacrifice. Mean is shown, n = 5 for GD2.CAR, n = 8 for GD2.CAR(I)IL12 and n = 9 for CD19.CAR(I)IL12, from three independent experiments; **p*=0.0114 CD19.CAR(I)IL12 vs. GD2.CAR(I)IL12; **p*=0.0269 GD2.CAR vs. GD2.CAR(I)IL12; ***p*=0.0080; unpaired t test. Source data are provided as a Source Data file. **D.** Quantification of human NKTs (iNKT$^+$CD45$^+$) in spleens collected at the time of sacrifice. Mean is shown, n = 5 for GD2.CAR, n = 8 for GD2.CAR(I)IL12 and n = 9 for CD19.CAR(I)IL12, from three independent experiments; **p*=0.0419 CD19.CAR(I)IL12 vs. GD2.CAR(I)IL12; **p*=0.0496 GD2.CAR vs. GD2.CAR(I)IL12; ***p*=0.0082; unpaired t test. Source data are provided as a Source Data file. **E.** Summary of CD62L expression in human NKTs (iNKT$^+$CD45$^+$) at sacrifice. Source data are provided as a Source Data file. **F.** Quantification of human cytokines detected in the serum of mice 4 weeks after the injection of CD19.CAR(I)IL12, GD2.CAR and GD2.CAR(I)IL12 NKTs. Mean is shown, n = 10; *****p*<0.0001; IL-4: ****p*=0.0009, IL-6: ****p*=0.0008, IL-10: ***p*=0.0025, ****p*=0.0009, TNF-α: ***p*=0.0054 GD2.CAR vs. CD19.CAR(I)IL12, ***p*=0.0042 GD2.CAR(I)IL12 vs. CD19.CAR(I)IL12; paired t test. Source data are provided as a Source Data file.

Figure S9

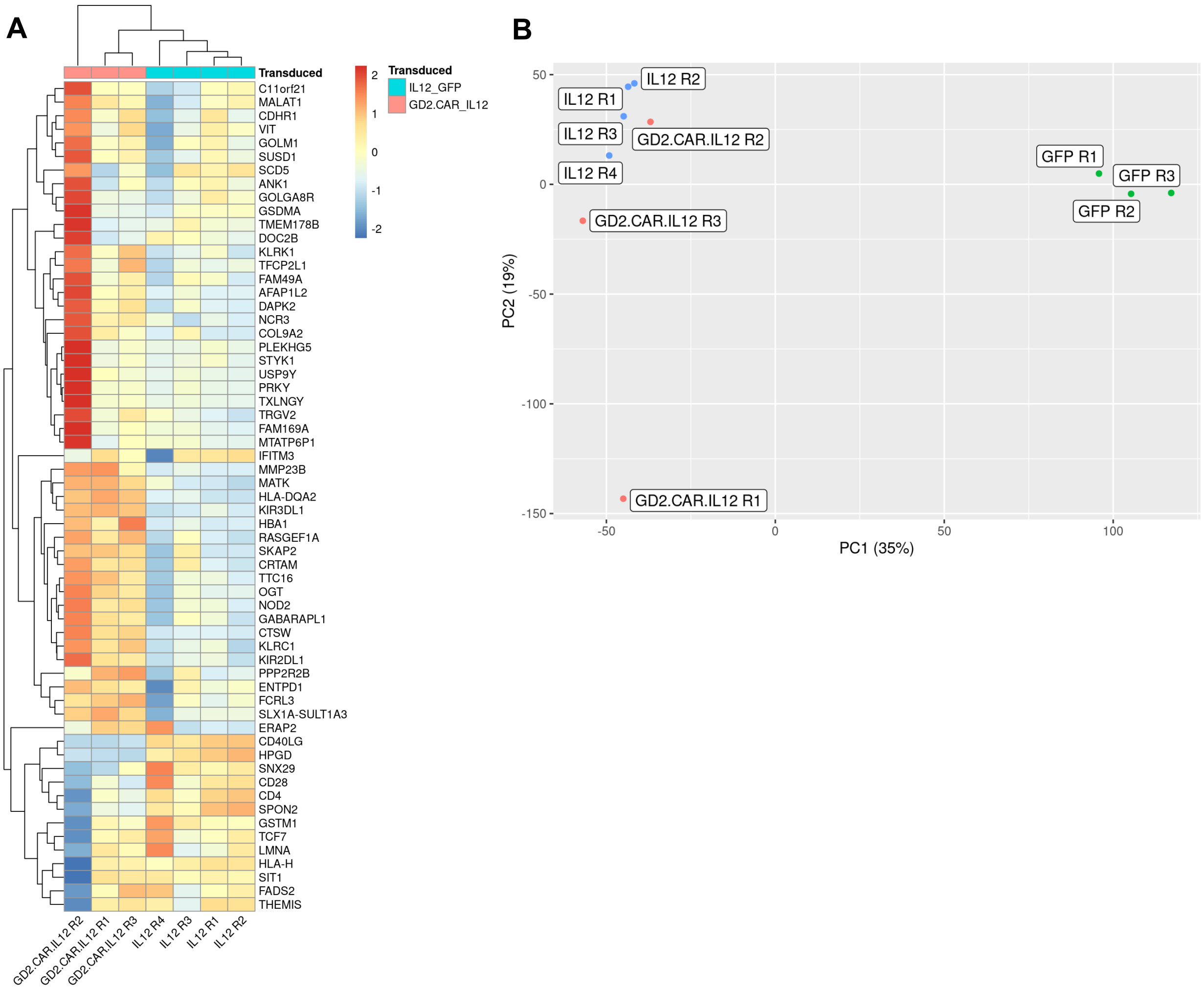

Figure S9. Gene expression profiles in IL12(I)GFP NKTs and GD2.CAR(I)IL12 NKTs. A. Heatmap of RNAseq differential gene expression between IL12(I)GFP and GD2.CAR(I)IL12 NKTs. All genes with an absolute log2 fold-change > 1 and an FDR-adjusted p-value <0.05 are included. **B.** Principal component analysis of RNAseq expression data from GFP, IL12(I)GFP and GD2.CAR(I)IL12 NKT samples.

Figure S10

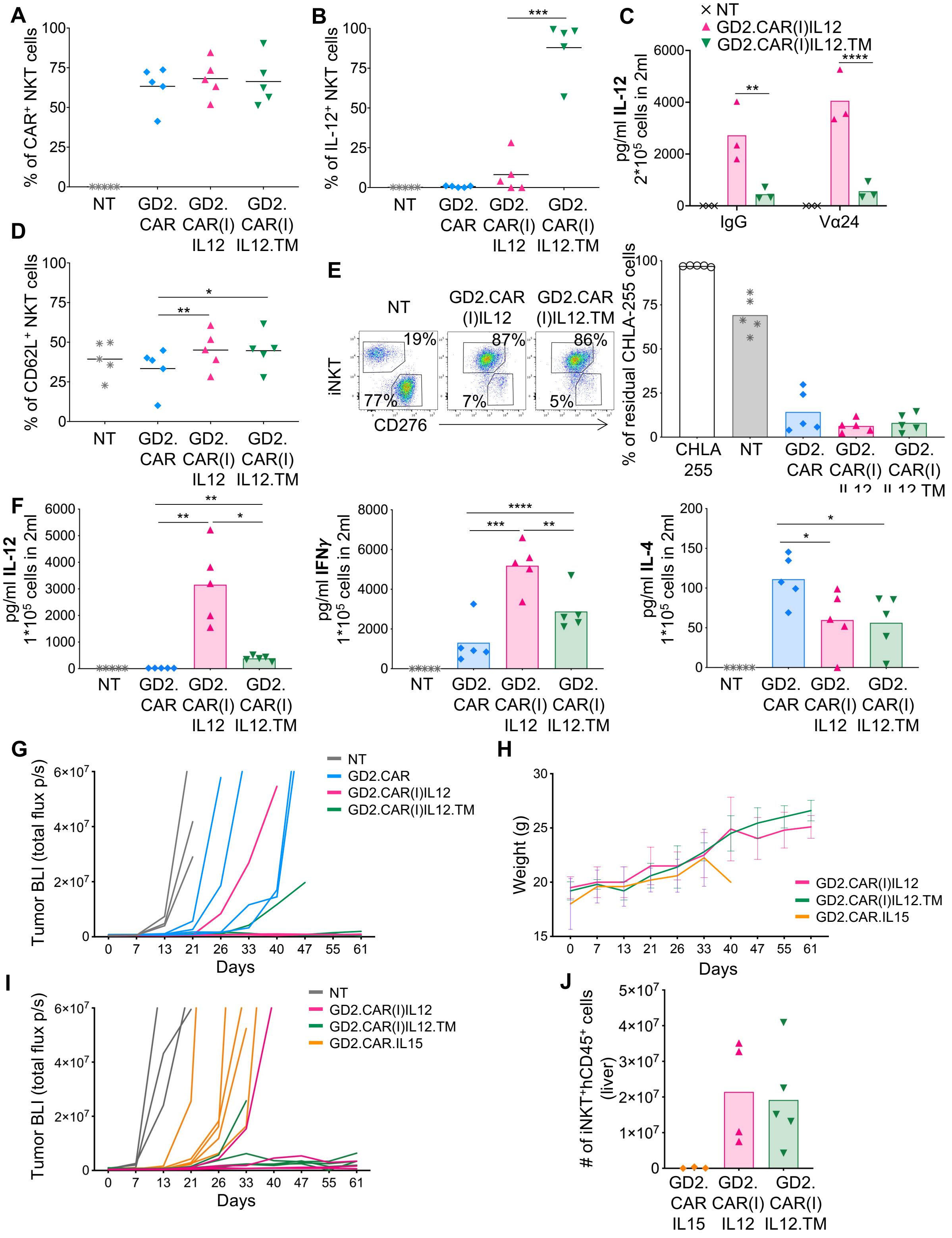

A
% of CAR+ NKT cells
NT
GD2. CAR
GD2. CAR(I) IL12
GD2. CAR(I) IL12.TM
B
% of IL-12+ NKT cells
C
NT
GD2.CAR(I)IL12
GD2.CAR(I)IL12.TM
pg/ml IL-12 2*10^5 cells in 2ml
IgG
Vα24
D
% of CD62L+ NKT cells
E
NT
GD2.CAR (I)IL12
GD2.CAR (I)IL12.TM
iNKT
CD276
19%
77%
87%
7%
86%
5%
% of residual CHLA-255 cells
CHLA 255
F
pg/ml IL-12 1*10^5 cells in 2ml
pg/ml IFNγ 1*10^5 cells in 2ml
pg/ml IL-4 1*10^5 cells in 2ml
G
Tumor BLI (total flux p/s)
Days
GD2.CAR
H
Weight (g)
GD2.CAR.IL15
I
J
# of iNKT+hCD45+ cells (liver)
GD2. CAR IL15

Figure S10. NKTs expressing the membrane-bound IL-12 upregulate CD62L and acquire pro-inflammatory properties. A. Summary of the CAR expression in control (NT) and transduced NKTs assessed at day 14 of culture. Mean is shown, n = 5. Source data are provided as a Source Data file. **B.** Summary of IL-12 expression on the cell surface of NT and transduced NKTs assessed at day 14 of culture. Mean is shown, n = 5; ****p*=0.0004; paired t test. Source data are provided as a Source Data file. **C.** Quantification of human IL-12 in supernatants collected 24 hrs after activation of NT, GD2.CAR(I)IL12 and GD2.CAR(I)IL12.TM with the iNKT Ab. IgG Ab was used as negative control. Cytokines were measured in supernatants collected 24 hrs in experiments in which 1 x 10^5 cells/well were plated in 24 well plates in 2 mL of complete media without cytokines. Mean is shown, n = 3; ** *p*=0.0012, **** *p*<0.0001; two-way ANOVA. Source data are provided as a Source Data file. **D.** Summary of the CD62L expression in NT and transduced NKTs assessed at day 14 of culture. Mean is shown, n = 5; **p*=0.0143; ***p*=0.0018; paired t test. Source data are provided as a Source Data file. **E.** Representative flow cytometry plots, and quantification of residual tumor cells in coculture experiments of CHLA-255 and NKTs (E:T=1:2). At day 4 cells were collected and stained with iNKT and CD276 Abs to identify NKTs and neuroblastoma cells, respectively, by flow cytometry. Source data are provided as a Source Data file. **F.** Quantification of IL-12, IFN-γ, and IL-4 in supernatants collected after 24 hrs from the coculture of CHLA-255 and NKTs (1 x 10^5 cells/well in 24 well plates in 2 mL of complete media without cytokines). Mean is shown, n = 5; **p*<0.0444; ***p*<0.0091; ****p*=0.0009; *****p*<0.0001; two-way ANOVA. Source data are provided as a Source Data file. **G.** Metastatic xenograft neuroblastoma model in which mice were engrafted i.v. with 1 x 10^6 CHLA-255 Firefly-luciferase labeled neuroblastoma tumor cells and treated with 5 x 10^6 NT, GD2.CAR, GD2.CAR(I)IL12 and GD2.CAR(I)IL12.TM NKTs. BLI tumor kinetics; n = 3 for NT and n = 4 for CAR NKTs. Source data are provided as a Source Data file. **H-J.** Metastatic xenograft neuroblastoma model in which mice were engrafted i.v. with 1 x 10^6 CHLA-255 Firefly-luciferase labeled neuroblastoma tumor cells and treated with 5 x 10^6 GD2.CAR.IL15 or GD2.CAR(I)IL12 or GD2.CAR(I)IL12.TM NKTs. Source data are provided as a Source Data file. **H.** Mice weight in grams. Mean and standard deviation are shown, n = 4 for GD2.CAR(I)IL12 and n = 5 for GD2.CAR(I)IL12.TM and GD2.CAR.IL15. **I.** BLI tumor kinetics; n = 3 for NT, n = 4 for GD2.CAR(I)IL12 and n = 5 for GD2.CAR(I)IL12.TM and GD2.CAR.IL15. GD2.CAR(I)IL12 vs. GD2.CAR.IL15 *p*=0.0016; GD2.CAR(I)IL12.TM vs. GD2.CAR.IL15 *p*=0.0007; two-way ANOVA. **J.** Quantification of human NKTs ($iNKT^+CD45^+$) in livers collected at the time of sacrifice. Mean is shown, n = 3 for GD2.CAR.IL15, n = 4 for GD2.CAR(I)IL12 and n = 5 for GD2.CAR(I)IL12.TM.

Figure S11

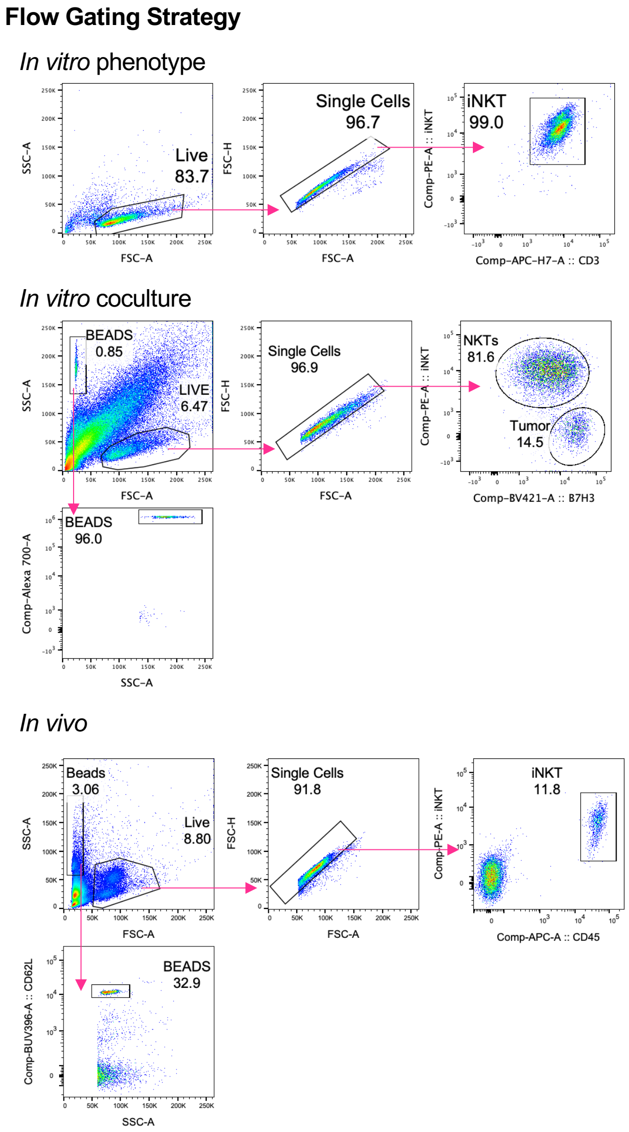

Figure S11. Flow gating strategy

Supplementary Reference

1. Diaconu I, Ballard B, Zhang M et al. Inducible Caspase-9 Selectively Modulates the Toxicities of CD19-Specific Chimeric Antigen Receptor-Modified T Cells. Mol.Ther. 2017;25:580-592.
