# Supplementary material for: IL-12 reprograms CAR-expressing natural killer T cells to long-lived Th1-polarized cells with potent antitumor activity
Source: Nat Commun. 2024 Jan 2;15:89. doi: 10.1038/s41467-023-44310-y (PMC10762263; doi:10.1038/s41467-023-44310-y)
Supplement: Supplementary file 3 — Reporting Summary [file 41467_2023_44310_MOESM3_ESM.pdf]

nature portfolio

Corresponding author(s): Gianpietro Dotti

Last updated by author(s): 11/30/2023

Reporting Summary

Nature Portfolio wishes to improve the reproducibility of the work that we publish. This form provides structure for consistency and transparency in reporting. For further information on Nature Portfolio policies, see our Editorial Policies and the Editorial Policy Checklist.

Statistics

For all statistical analyses, confirm that the following items are present in the figure legend, table legend, main text, or Methods section.

n/a	Confirmed	
☐	☒	The exact sample size (*n*) for each experimental group/condition, given as a discrete number and unit of measurement
☐	☒	A statement on whether measurements were taken from distinct samples or whether the same sample was measured repeatedly
☐	☒	The statistical test(s) used AND whether they are one- or two-sided<br>*Only common tests should be described solely by name; describe more complex techniques in the Methods section.*
☐	☒	A description of all covariates tested
☐	☒	A description of any assumptions or corrections, such as tests of normality and adjustment for multiple comparisons
☐	☒	A full description of the statistical parameters including central tendency (e.g. means) or other basic estimates (e.g. regression coefficient) AND variation (e.g. standard deviation) or associated estimates of uncertainty (e.g. confidence intervals)
☐	☒	For null hypothesis testing, the test statistic (e.g. *F*, *t*, *r*) with confidence intervals, effect sizes, degrees of freedom and *P* value noted<br>*Give P values as exact values whenever suitable.*
☒	☐	For Bayesian analysis, information on the choice of priors and Markov chain Monte Carlo settings
☒	☐	For hierarchical and complex designs, identification of the appropriate level for tests and full reporting of outcomes
☒	☐	Estimates of effect sizes (e.g. Cohen's *d*, Pearson's *r*), indicating how they were calculated

Our web collection on statistics for biologists contains articles on many of the points above.

Software and code

Policy information about availability of computer code

Data collection	Flow cytometry data were collected on BD LSR Fortessa and BD Canto II using BD FACS DIVA software.<br>qPCR data acquisition was performed on a QuantStudio 6 Flex from Life Technologies.<br>Polifunctionality data were acquired on the isolight from isoplexis.<br>ELISA data were acquired on the Bio-Plex 200 System (BIO-RAD)<br>In vivo bioluminescence date were acquired on IVIS kinetic in vivo imaging system (PerkinElmer)
Data analysis	Flow cytometry data was analyzed using Flowjo v10. GraphPad Prism 8 is a graphing software that was used for figure generation and statistical analyses.<br>qPCR data were analyzed with QuantStudio Real-Time PCR software<br>Polifunctionality data were analyzed using the IsoSpeak (v2.9.0) software.<br>ELISA data were analyzed with the Bio-Plex Manager 6.1 software (Bio-Rad)<br>In vivo bioluminescence date were acquired on living image (V4.3.1) software<br>RNA- seq data were aligned with STAR alignment (v.2.4.2) and quantified with Salmon (v.0.6.0). Paired- end FASTQ files were aligned to an Ensembl transcriptome (release 99, on reference genome GRCh38) using Star (v2.7.6a) and transcripts quantified using Salmon (v1.4.0). Quality of FASTQ data and quantified BAMs was verified using FastQC (v0.11.9) and Picard's (v2.22.4) CollectRnaSeqMetrics program, respectively. Differential gene expression was calculated and compared in R (v4.1.1) using the DESeq2 (v1.34.0) Bioconductor package.

For manuscripts utilizing custom algorithms or software that are central to the research but not yet described in published literature, software must be made available to editors and reviewers. We strongly encourage code deposition in a community repository (e.g. GitHub). See the Nature Portfolio guidelines for submitting code & software for further information.

Data

Policy information about availability of data

All manuscripts must include a data availability statement. This statement should provide the following information, where applicable:

- Accession codes, unique identifiers, or web links for publicly available datasets
- A description of any restrictions on data availability
- For clinical datasets or third party data, please ensure that the statement adheres to our policy

RNA Seq data that support the findings of this study have been deposited in GEO with the accession code GSE241586.
https://www.ncbi.nlm.nih.gov/geo/query/acc.cgi?acc=GSE241586

Research involving human participants, their data, or biological material

Policy information about studies with human participants or human data. See also policy information about sex, gender (identity/presentation), and sexual orientation and race, ethnicity and racism.

Reporting on sex and gender	na
Reporting on race, ethnicity, or other socially relevant groupings	na
Population characteristics	na
Recruitment	na
Ethics oversight	na

Note that full information on the approval of the study protocol must also be provided in the manuscript.

Field-specific reporting

Please select the one below that is the best fit for your research. If you are not sure, read the appropriate sections before making your selection.

☒ Life sciences ☐ Behavioural & social sciences ☐ Ecological, evolutionary & environmental sciences

For a reference copy of the document with all sections, see nature.com/documents/nr-reporting-summary-flat.pdf

Life sciences study design

All studies must disclose on these points even when the disclosure is negative.

Sample size	Sample sizes for experiments was decided to obtain a statistical power higher than 80% and based on previous experience (Hirabayashi, K., Du, H., Xu, Y., Shou, P., Zhou, X., Fucá, G., Landoni, E., Sun, C., Chen, Y., Savoldo, B., & Dotti, G. (2021). Dual Targeting CAR-T Cells with Optimal Costimulation and Metabolic Fitness enhance Antitumor Activity and Prevent Escape in Solid Tumors. Nature cancer, 2(9), 904–918. https://doi.org/10.1038/s43018-021-00244-2).<br>All in vitro experiments were repeated at least three separate times (three different NKT cell donors). For in vivo experiments using NSG mice, all experiments were repeated using at least two different NKT cell donors with at 2-4 mice in each group (4-8 mice/group in total).
Data exclusions	No data was excluded.
Replication	All experiments included showed similar trend in repeated attempts. Experiment were replicated between 2 and 10 times, everytime using different donors.
Randomization	Samples and organisms were randomly allocated to groups for in vitro and in vivo experiments.
Blinding	No formal blinding was used since it is not feasible with multiple treatment groups and multiple steps of sample preparation.

Reporting for specific materials, systems and methods

We require information from authors about some types of materials, experimental systems and methods used in many studies. Here, indicate whether each material, system or method listed is relevant to your study. If you are not sure if a list item applies to your research, read the appropriate section before selecting a response.

Materials & experimental systems

n/a	Involved in the study
☐	☒ Antibodies
☐	☒ Eukaryotic cell lines
☒	☐ Palaeontology and archaeology
☐	☒ Animals and other organisms
☒	☐ Clinical data
☒	☐ Dual use research of concern
☒	☐ Plants

Methods

n/a	Involved in the study
☒	☐ ChIP-seq
☐	☒ Flow cytometry
☒	☐ MRI-based neuroimaging

Antibodies

Antibodies used	NKTs were stained with antibodies (Ab) against CD3 (APC-H7, clone SK7), CD62L (BV605, clone DREG-56), CD4 (PE-Cy7, clone SK3), CD8 (Alexa Fluor 700, clone RPA-T8), CD19 (APC, clone HIB19), CD45 (APC, clone 2D1), CD271 (NGFR, APC, clone C40- 1457), CD279 (PD-1, PE-Cy7, clone EH12.1), CD366 (TIM-3, BV711, clone 7D3), CD223 (LAG- 3, PE, clone T47-530) and CD152 (CTLA-4, BV421, clone BNI3) from BD Biosciences; IL-12 (p70, APC) and CD212 (IL12R b2, APC) from Miltenyi Biotech. Tumor cells were stained with Abs against GD2 (PE, clone 14.G2a) and CD276 (B7-H3, BV421, clone 7-517) from BD Biosciences. The purity of NKTs was assessed by staining the cells with the PE-conjugated Abs specific for TCR Vα24 chain (iNKT, clone 6B11, BD Biosciences) or for TCR β11 chain (FITC, Beckman Coulter), which we have previously shown to be superimposable. The expression of the CD19.CAR, GD2.CAR and CSPG4.CAR was assessed using specific anti-idyotipic Abs, followed by the staining with a secondary goat anti-Mouse Ab (APC, Ig multiple adsorption, BD Biosciences).<br>For westenblot the following Abs were used: α-Stat4 (C46B10, dilution 1:1000) and α-Phospho-Stat4 (Tyr693, dilution 1:1000) from Cell Signaling; α-CD3ζ (dilution 1:1000) horseradish peroxidase (HRP) conjugated from Santa Cruz; HRP conjugated secondary Ab (Goat-α-Rabbit #32460, dilution 1:500) from Thermo Scientific.
Validation	All flow cytomety and westen blot antibodies were validated with negative and positive cell line or related isotype ctrl antibodies.<br>Primaries antibodies used to detect the CAR were previously validated<br>GD2 CAR: Chen, Y., Sun, C., Landoni, E., Metelitsa, L., Dotti, G., & Savoldo, B. (2019). Eradication of Neuroblastoma by T Cells Redirected with an Optimized GD2-Specific Chimeric Antigen Receptor and Interleukin-15. Clinical cancer research : an official journal of the American Association for Cancer Research, 25(9), 2915–2924. https://doi.org/10.1158/1078-0432.CCR-18-1811<br>CD19 CAR: Ramos, C. A., Rouce, R., Robertson, C. S., Reyna, A., Narala, N., Vyas, G., Mehta, B., Zhang, H., Dakhova, O., Carrum, G., Kamble, R. T., Gee, A. P., Mei, Z., Wu, M. F., Liu, H., Grilley, B., Rooney, C. M., Heslop, H. E., Brenner, M. K., Savoldo, B., ... Dotti, G. (2018). In Vivo Fate and Activity of Second- versus Third-Generation CD19-Specific CAR-T Cells in B Cell Non-Hodgkin's Lymphomas. Molecular therapy : the journal of the American Society of Gene Therapy, 26(12), 2727–2737. https://doi.org/10.1016/j.ymthe.2018.09.009

Eukaryotic cell lines

Policy information about cell lines and Sex and Gender in Research

Cell line source(s)	B cell leukemia cell line BV-173 was purchased from DSMZ and Burkitt's lymphoma cell line Daudi was purchased from ATCC. Human neuroblastoma (NB) cell lines CHLA-255 and firefly luciferase-labeled (FFLuc)-CHLA-255 were gifts from Dr. Leonid Metelitsa at Baylor College of Medicine (originally derived from a metastatic lesion in the brain in a patient with recurrent disease at Children's Hospital Los Angeles) and LAN-1 was a gift from Dr. Malcom Brenner at Baylor College of Medicine, originally purchased from ATCC.
Authentication	All cell lines were routinely tested for cell surface markers and with PCR assays with species-specific primers
Mycoplasma contamination	All cell lines were routinely tested for mycoplasma and were always negative.
Commonly misidentified lines (See ICLAC register)	None of cell line used are commonly misidentified lines based on ICLAC register V9.

Animals and other research organisms

Policy information about studies involving animals; ARRIVE guidelines recommended for reporting animal research, and Sex and Gender in Research

Laboratory animals	7- 9-week-old wild type NSG mice (UNCCH in-house breeding).<br>Mice are in sterile housing- (autoclaved cages and food) and RO, purified water.<br>12 hours alternating dark and light.<br>ambient temperature 68-74 degrees in the animal rooms.<br>humidity 30-70 degrees
Wild animals	na
Reporting on sex	na

Field-collected samples	na
Ethics oversight	Animal experiments were performed according to IACUC-approved protocols at University of North Carolina.

Note that full information on the approval of the study protocol must also be provided in the manuscript.

Flow Cytometry

Plots

Confirm that:

- [x] The axis labels state the marker and fluorochrome used (e.g. CD4-FITC).
- [x] The axis scales are clearly visible. Include numbers along axes only for bottom left plot of group (a 'group' is an analysis of identical markers).
- [x] All plots are contour plots with outliers or pseudocolor plots.
- [x] A numerical value for number of cells or percentage (with statistics) is provided.

Methodology

Sample preparation	For surface staining, cells were incubated with antibodies at room temperature for 15 min or at refrigerator for 20 min.
Instrument	Flow cytometry data were collected on BD LSR Fortessa and BD Canto II using BD FACS DIVA software.
Software	BD FACS DIVA software and Flowjo software
Cell population abundance	In one experiment NKTs were sorted for CD62L expresion, The post sort purity was above 80% in the positive fraction detected by flow post-sorting
Gating strategy	1. Gating live cells based on physical parameters FSC-A vs SSC.<br>2. On the live cell gating for single cells FSC-A vs FSC-H<br>3. All other gaiting.

- [ ] Tick this box to confirm that a figure exemplifying the gating strategy is provided in the Supplementary Information.
